# Long-Term Effects of Statin Treatment in Elderly People: Extended Follow-Up of the PROspective Study of Pravastatin in the Elderly at Risk (PROSPER)

**DOI:** 10.1371/journal.pone.0072642

**Published:** 2013-09-02

**Authors:** Suzanne M. Lloyd, David J. Stott, Anton J. M. de Craen, Patricia M. Kearney, Naveed Sattar, Ivan Perry, Christopher J. Packard, Andrew Briggs, Laura Marchbank, Harry Comber, J. Wouter Jukema, Rudi G. J. Westendorp, Stella Trompet, Brendan M. Buckley, Ian Ford

**Affiliations:** 1 Robertson Centre for Biostatistics, University of Glasgow, Glasgow, United Kingdom; 2 Institute of Cardiovascular and Medical Sciences, University of Glasgow, Glasgow, United Kingdom; 3 Department of Gerontology and Geriatrics, Leiden University Medical Center, Leiden, The Netherlands; 4 Department of Epidemiology and Public Health, University College, Cork, Ireland; 5 National Health Service Research and Development Directorate, Greater Glasgow and Clyde Health Board, Glasgow, United Kingdom; 6 Health Economics and Health Technology Assessment, University of Glasgow, Glasgow, United Kingdom; 7 Information Services Division Scotland, National Health Service (Scotland), Paisley, United Kingdom; 8 National Cancer Registry, Cork, Ireland; 9 Department of Cardiology, Leiden University Medical Center, Leiden, The Netherlands; 10 Department of Pharmacology and Therapeutics, University College, Cork, Ireland; Innsbruck Medical University, Austria

## Abstract

**Background:**

The PROspective Study of Pravastatin in the Elderly at Risk (PROSPER), a placebo-controlled trial of pravastatin, demonstrated a 19% reduction in coronary outcomes (p = 0.006) after a mean of 3.2 years, with no impact on stroke outcomes or all-cause mortality. However, there was a suggestion of increased cancer risk. Our aim is to determine the long-term benefits and safety of pravastatin treatment in older people using post-trial follow-up of the PROSPER participants.

**Methods:**

5,804 (2,520 Scottish) men and women aged 70–82 years with either pre-existing vascular disease or increased risk of such disease because of smoking, hypertension or diabetes, were randomised to 40 mg pravastatin or matching placebo. Using record linkage to routinely collected health records, all participants (full cohort) were linked to death and cancer registries, and the Scottish cohort additionally to hospital admissions, to provide composite fatal/non-fatal cardiovascular outcomes (total mean follow-up 8.6 years).

**Results:**

Pravastatin treatment for 3.2 years reduced CHD death in the full cohort, hazard ratio (HR) 0.80, 95% confidence interval (CI) 0.68–0.95, p = 0.0091 and fatal coronary events or coronary hospitalisations in the Scottish cohort (HR 0.81, 95% CI 0.69–0.95, p = 0.0081) over 8.6 years. There was no reduction in stroke or all-cause mortality. Cancer risk was not increased in the full cohort (HR 1.08, 95% CI 0.96–1.21, p = 0.22).

**Conclusions:**

Pravastatin treatment of elderly high-risk subjects for 3.2 years provided long-term protection against CHD events and CHD mortality. However, this was not associated with any increase in life expectancy, possibly due to competing mortality with deaths from other causes. There was no evidence of long-term increased risk of cancer.

**Trial registration:**

ISRCTN40976937.

## Introduction

Ischaemic vascular disease is a major contributor to death and disease in older age. Statins are now widely used in elderly people, with the aim of preventing ill health and extending life. However, the evidence for vascular benefit from these drugs in older people is over a relatively short term of a few years, and critically it is not yet known whether such benefits are sustained, are associated with prolonged life expectancy or whether any hazard emerges.

The PROspective Study of Pravastatin in the Elderly at Risk (PROSPER) was a clinical trial in which 5804 men (n = 2804) and women (n = 3000) aged 70–82 years with a history of, or risk factors for, vascular disease were randomised to treatment with pravastatin (40 mg per day) or placebo. Pravastatin lowered low density lipoprotein cholesterol (LDL-cholesterol) concentrations by 34%. After an average follow-up of 3.2 years the primary endpoint, a composite of coronary death, non-fatal myocardial infarction (MI), and fatal or non-fatal stroke was reduced by 15% (hazard ratio [HR] 0.85, 95% Confidence Interval [CI] 0.74–0.97, p = 0.014); benefit was limited to a reduction in coronary heart disease death or non-fatal myocardial infarction (HR 0.81, 95% CI 0.69–0.94, p = 0.006), with no effect on stroke or all cause mortality [1]–[3].

New cancer diagnoses were more frequent on pravastatin than on placebo (HR 1.25, 95% CI 1.04–1.51, p = 0.020). This was thought to be a chance finding and inclusion of these data in meta-analyses of randomised controlled trials of statins showed no overall increase in risk of cancer [3]–[5].

We have shown previously that extended post-trial follow-up can provide important additional information on the long-term benefits and safety of randomised treatment in the WOSCOPS trial [6]. PROSPER involved participants with and without a history of vascular disease. Recently, the treatment of patients in primary prevention has been questioned [7], while others have argued that we should treat more aggressively at a younger age [8]. In this paper we provide the results of the extended follow-up of PROSPER, examining the post-trial effects of a period of pravastatin treatment in older age.

## Methods

### Design

The design, baseline characteristics and results of PROSPER have been published elsewhere [1]–[3]. Between 1997 and 1999, we recruited men and women from Scotland, Ireland, and the Netherlands aged 70–82 if they had either pre-existing vascular disease (coronary, cerebral, or peripheral) or increased risk of such disease because of smoking, hypertension, or diabetes. Baseline plasma total cholesterol was in the range 4.0–9.0 mmol/L. The ethics committees of all centres approved the protocol (Research Ethics Committee of the Cork Teaching Hospitals (CREC), Scottish Multi-Regional Ethics Committee A and Medical Ethical Committee (METc) of the Leiden University Medical Center) and participants gave written informed consent for the trial and long-term follow-up. In addition record linkage in Scotland was approved by the Privacy Advisory Committee to the National Health Service (NHS) National Services Scotland and cancer linkage in Ireland and the Netherlands, respectively, was conducted by the Irish National Cancer Registry and the Dutch Cancer Registry.

### Endpoints and record linkage

The primary outcome of the original trial was CHD death, non-fatal MI or fatal or non-fatal stroke and endpoints were adjudicated by an endpoint adjudication committee.

The results presented in this paper are based entirely on computerised record linkage. In each country we were able to obtain information on cause-specific mortality and incident cancers, from linkage to mortality registries and regional or national cancer registries. Hence, we have data from all three countries for the investigation of the effects of statin treatment on mortality and cancer incidence.

In Scotland, in addition, we were able to link to hospital discharge summaries held by the Information Services Division of the National Health Service for Scotland by means of established record-linkage methods [9]. Hence, for the study of composite fatal or non fatal cardiovascular outcomes our cohort is restricted to Scottish participants.

During the original trial, 12 participants withdrew informed consent and hence their follow-up has been censored at the time of withdrawal of consent.

Data on outcome events were extracted from the databases using appropriate International Classification of Diseases (ICD) codes and information on operations and procedures using Office of Population, Censuses and Surveys Classification of Surgical Operations and Procedures (OPCS) codes. For deaths and hospital discharge summaries, data were available until 30^th^ June 2009 and for incident cancers until 31st December 2008.

Outcomes analysed for the full cohort were all-cause mortality, coronary, stroke, cancer and non-cardiovascular mortality and, for the Scottish cohort, the composite of death or hospitalisation due to MI or stroke, death due to stroke or hospitalisation due to stroke, coronary death or hospitalisation for MI and coronary death or coronary hospitalisation.

For inclusion as a hospitalisation outcome, MI or stroke was included whether or not it was coded as the primary reason for admission. For other coronary events to be counted they had to be listed as the principal reason for admission. Deaths were categorised based on the underlying cause of death on the death record. Incident cancers were identified from the cancer registries or from the cause of, or factors contributing to, death. Comparison of the record linkage cancers with those identified during the conduct of the trial revealed discrepancies between the coding of the events from the two sources, the main differences being associated with skin cancers and the classification of neoplasms as being benign, malignant or of uncertain or unknown behaviour. Hence, we report first incident cancers excluding these additional categories, then including minor skin cancers and finally including neoplasms coded as of uncertain or unknown behaviour.

### Statistical methods

Baseline characteristics were summarised as mean (standard deviation) for continuous variables and as count (percentage) for categorical variables. Years of follow-up were calculated from the date of randomisation until censoring due to end of follow-up period, withdrawal of consent or death, whichever came first. Cumulative incidence functions were calculated for outcomes taking into account the competing risk of non-included causes of death as appropriate. Cause-specific Cox proportional-hazards models were fitted including the original study-group assignment (pravastatin or placebo) and relevant baseline risk factors as reported previously [10]. Adjustment was made for age, sex, smoking status, body mass index (BMI), systolic and diastolic blood pressure (SBP & DBP), high density lipoprotein (HDL) and low density lipoprotein (LDL) cholesterol, and histories of diabetes, hypertension, coronary, cerebrovascular and peripheral vascular disease. Treatment effects (pravastatin compared with placebo) are expressed as hazard ratios with 95% confidence intervals and corresponding p-values. Treatment effects were estimated within the trial, in extended follow-up in those surviving the trial period, and over the entire period of follow-up. Although we thought it was possible that the proportional-hazards assumption would not be valid over the full period of follow-up for all analyses, we concluded that the estimated hazard ratios would still reflect an average benefit over the period. As there was no evidence of an impact of pravastatin treatment on stroke incidence during the trial, subgroup analyses were restricted to the most frequent coronary outcome, namely coronary disease death or coronary hospitalisation. Subgroup analyses were carried out in pre-defined subgroups based on a history of vascular disease, sex, thirds of the distributions of LDL- and HDL-cholesterol, current smoking and history of hypertension. While diabetes was a predefined subgroup, it was an infrequent reason for inclusion and hence there were inadequate data for this report.

For the incident cancer endpoint we had approximately 80% power to detect an increased risk of 30% (Hazard Ratio of 1.3) during the trial period, an increased risk of 24.5% (Hazard Ratio of 1.245) during the post-trial period and an increased risk of 18% (Hazard Ratio of 1.18) for the total follow-up period.

## Results

A total of 5804 participants were randomised, and 5188 survived and completed the trial and were followed up long-term. Corresponding figures for the Scottish participants were 2520 randomised and 2234 with extended follow-up. The average follow-up during the trial was 3.2 years (maximum 4.0 years) and 8.6 years over the entire follow-up (maximum 11.3 years). Baseline characteristics are given in Table S1 for the full and Scottish cohorts.

### Mortality (full cohort)

**Figure 1:**
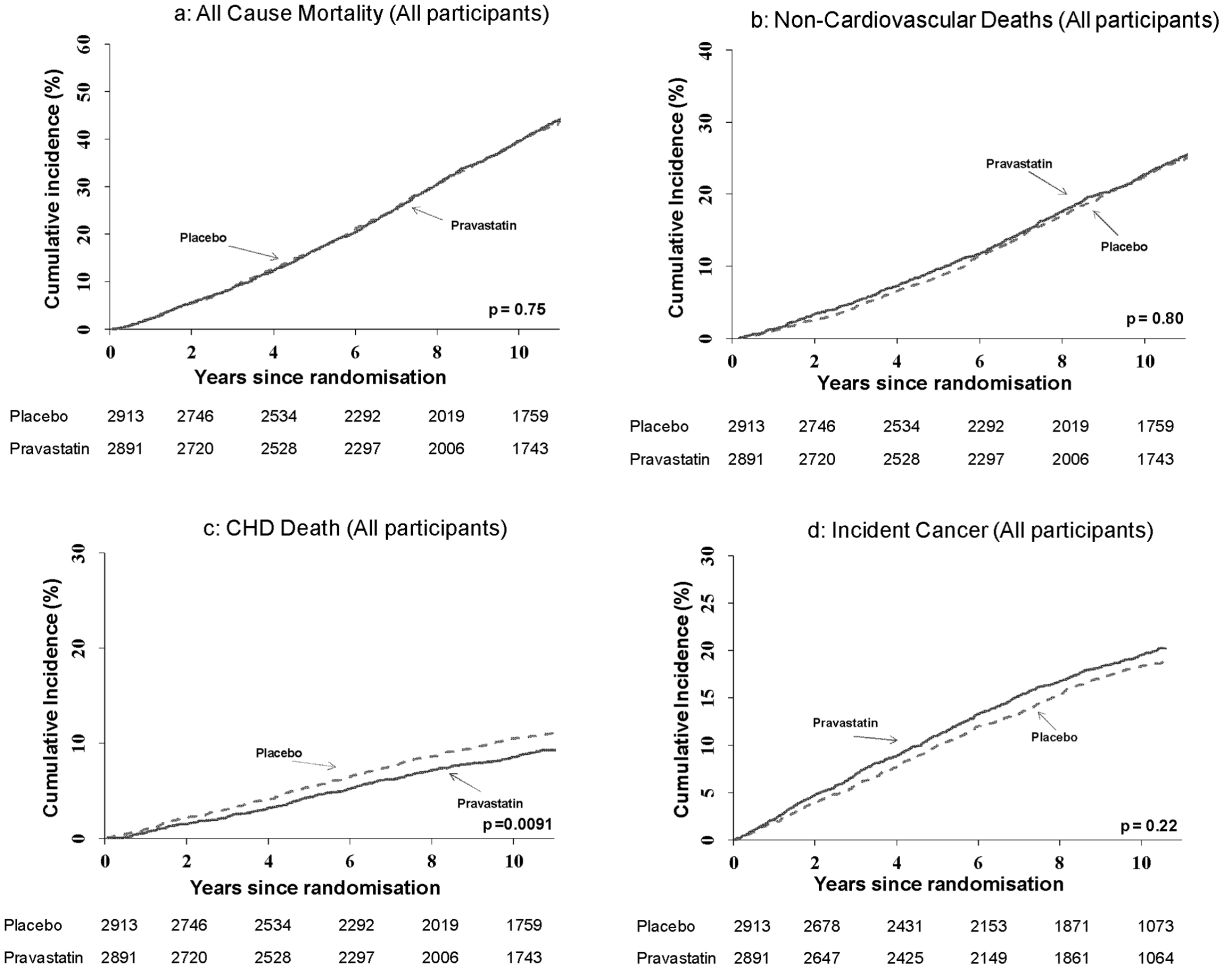
Cumulative incidence plots for all cause mortality (1a), non-cardiovascular deaths (1b), coronary heart disease (CHD) deaths (1c) and incident cancers (1d) for the full cohort. Numbers at risk are presented for each treatment group.

Table 1 gives the overall estimated treatment effects for all cause mortality and non-cancer mortality. The cumulative incidence functions for all-cause, non-cardiovascular and coronary deaths are given in Figures 1a, 1b and 1c. There was no evidence of any effect on all-cause mortality or on non-cardiovascular or cardiovascular mortality. During the trial and post-trial there was a numerical excess of stroke deaths in the pravastatin arm. However, this difference did not reach statistical significance. There was a reduction in coronary heart disease mortality over the entire period of follow-up (HR 0.80, 95% CI 0.68–0.95, p = 0.0091).

**Table 1:** Mortality outcomes in the full cohort: participants and deaths by randomized treatment group, hazard ratios (95% CIs) and p-values within the trial, in the post-trial period and overall.

*Cause of Death*	*Trial Period*		*Post-Trial Period*		*Total Follow-up*	
	*Placebo*	*Pravastatin*	*Placebo*	*Pravastatin*	*Placebo*	*Pravastatin*
	*(N = 2913)*	*(N = 2891)*	*(N = 2600)*	*(N = 2588)*	*(N = 2913)*	*(N = 2891)*
**All causes**						
Deaths–no (%)	306 (10·5%)	298 (10·3%)	928 (35·7%)	931 (36·0%)	1234 (42·4%)	1229 (42·5%)
Hazard ratio (95% CI)	Referent	0·97 (0·83–1·14)	Referent	0·99 (0·91–1·09)	Referent	0·99 (0·91–1·07)
p-value		0·70		0·88		0·75
**Non-cardiovascular death**						
Deaths–no (%)	152 (5·2%)	176 (6·1%)	553 (21·3%)	535 (20·7%)	705 (24·2%)	711 (24·6%)
Hazard ratio (95% CI)	Referent	1·18 (0·95–1·46)	Referent	0·97 (0·86–1·09)	Referent	1·01 (0·91–1·12)
p-value		0·14		0·60		0·80
**Cardiovascular**						
Deaths–no (%)	154 (5·3%)	122 (4·2%)	375 (14·4%)	396 (15·3%)	529 (18·2%)	518 (17.9%)
Hazard ratio (95% CI)	Referent	0·77 (0·61–0·98)	Referent	1·03 (0·89–1·18)	Referent	0·95 (0·84–1·08)
p-value		0·033		0·71		0·43
**CHD**						
Deaths–no (%)	102 (3·5%)	79 (2·7%)	216 (8·3%)	185 (7·1%)	318 (10.9%)	264 (9·1%)
Hazard ratio (95% CI)	Referent	0·75 (0·56–1·00)	Referent	0·83 (0·68–1·01)	Referent	0·80 (0·68–0·95)
p-value		0·052		0·069		0·0091
**Stroke**						
Deaths–no (%)	16 (0·5%)	19 (0·7%)	84 (3·2%)	109 (4·2%)	100 (3·4%)	128 (4·4%)
Hazard ratio (95% CI)	Referent	1·23 (0·63–2·40)	Referent	1·25 (0·94–1·66)	Referent	1·24 (0·96–1·62)
p-value		0·54		0·13		0·10

### Cancers (full cohort)

Results for cancer mortality and incident cancers are given in Table 2 with the cumulative incidence function for incident cancer in Figure 1d. There was no evidence of an increase in cancer mortality on statin treatment. A suggestion of an increased risk of incident cancer during the trial period (HR 1.23, 95% CI 1.01–1.49, p = 0.038) was not replicated in the post-trial period, HR 1.08, 95% CI 0.96–1.21, p = 0.22 for the entire period of follow-up.

**Table 2:** Cancer mortality and incident cancer outcomes in the full cohort: participants and participants with events by randomized treatment group, hazard ratios (95% CIs) and p-values within the trial, in the post-trial period and overall.

	*Trial Period*		*Post-Trial Period*		*Total Follow-up*		
*Endpoint*	*Placebo*	*Pravastatin*	*Placebo*	*Pravastatin*	*Placebo*	*Pravastatin*	
**Incident Cancer**	N = 2913	N = 2891	N = 2543	N = 2501	N = 2913	N = 2891	
Events–no (%)	191 (6·6%)	230 (8·0%)	346 (13·6%)	340 (13·6%)	537 (18·4%)		570 (19·7%)
Hazard ratio (95% CI)	Referent	1·23 (1·01–1·49)	Referent	1·00 (0·86–1·16)	Referent		1·08 (0·96–1·21)
p-value		0·038		0·95			0·22
**Cancer Deaths**	N = 2913	N = 2891	N = 2600	N = 2588	N = 2913	N = 2891	
Events–no (%)	87 (3·0%)	118 (4·1%)	236 (9·1%)	236 (9·1%)	323 (11·1%)		354 (12·2%)
Hazard ratio (95% CI)	Referent	1·37 (1·04–1·81)	Referent	1·02 (0·85–1·22)	Referent		1·12 (0·96–1·30)
p-value		0·026		0·85			0·15
Incident Cancer*	N = 2913	N = 2891	N = 2486	N = 2445	N = 2913	N = 2891	
Events–no (%)	250 (8·6%)	291 (10·1%)	463 (18·6%)	439 (18·0%)	713 (24·5%)		730 (25·3%)
Hazard ratio (95% CI)	Referent	1·18 (0·99–1·39)	Referent	0·96 (0·84–1·09)	Referent		1·03 (0·93–1·15)
p-value		0·060		0·53			0·52
Incident Cancer**	N = 2913	N = 2891	N = 2480	N = 2430	N = 2913	N = 2891	
Events–no (%)	265 (9·1%)	311 (10·8%)	489 (19·8%)	467 (19·3%)	754 (25·9%)		778 (26·9%)
Hazard ratio (95% CI)	Referent	1·19 (1·01–1·40)	Referent	0·97 (0·85–1·10)	Referent		1·04 (0·94–1·15)
p-value		0·041		0·62			0·40

Incident cancer results are repeated including minor skin cancers* and neoplasms of uncertain or unknown behaviour**.

### Composite fatal and non-fatal cardiovascular outcomes (Scottish cohort only)

**Figure 2:**
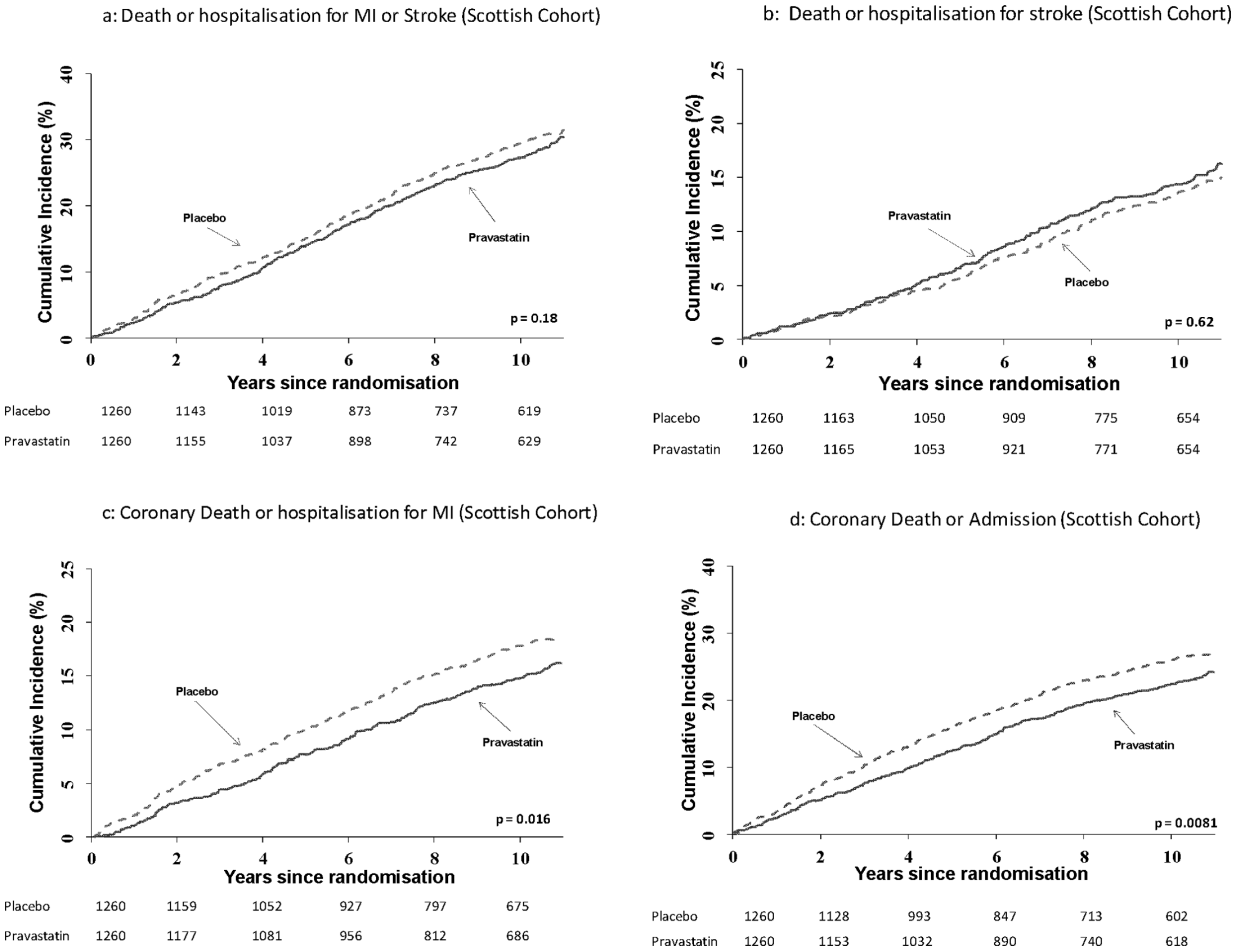
Cumulative incidence plots for the combined endpoints of death or hospitalization for myocardial infarction (MI) or stroke (2a), death or hospitalization for stroke (2b), coronary deaths or hospitalizations for MI (2c) and coronary death or admission (2d) for the Scottish cohort. Numbers at risk are presented for each treatment group.

Results for fatal and non-fatal cardiovascular outcomes are given in Table 3 and illustrated in Figure 2. There was no evidence of reduction in stroke events on pravastatin, nor did the results for the composite of coronary or stroke death or stroke or MI hospitalization reach statistical significance. However, there was evidence of a reduction in CHD death or non-fatal MI within-trial (HR 0.64, 95% CI 0.46–0.88, p = 0.007) and overall (HR 0.79, 95% CI 0.66–0.96, p = 0.016), with corresponding results for the outcome of CHD death or coronary hospitalization (HR 0.81, 95% CI 0.69–0.95, p = 0.0081) in the long-term follow-up. Coronary revascularisation (coronary artery bypass graft or percutaneous coronary intervention) was uncommon in this elderly population with similar numbers of events in both groups (33 (2.6%) on placebo and 30 (2.4%) on pravastatin).

**Table 3:** Composite cardiovascular outcomes in the Scottish cohort: results contain participants and participants with events by randomized treatment group, hazard ratios (95% CIs) and p-values within the trial, in the post-trial period and overall.

	*Trial Period*			*Post-Trial Period*		*Total Follow-up*	
*Event*	*Placebo*		*Pravastatin*	*Placebo*	*Pravastatin*	*Placebo*	*Pravastatin*
**CHD or stroke death or MI or stroke admission**	N = 1260		N = 1260	N = 1050	N = 1071	N = 1260	N = 1260
Events–no (%)	135 (10·7%)	113 (9·0%)		256 (24·4%)	256 (23·9%)	391 (31·0%)	369 (29·3%)
Hazard ratio (95% CI)	Referent	0·80 (0·62–1·03)		Referent	0·96 (0·81–1·15)	Referent	0·91 (0·79–1·05)
p-value		0·088			0·67		0·18
**Stroke death or stroke admission**	N = 1260	N = 1260		N = 1081	N = 1082	N = 1260	N = 1260
Events–no (%)	50 (4·0%)	56 (4·4%)		135 (12·5%)	141 (13·0%)	185 (14·7%)	197 (15·6%)
Hazard ratio (95% CI)	Referent	1·11 (0·76–1·63)		Referent	1·03 (0·81–1·31)	Referent	1·05 (0·86–1·29)
p-value		0·59			0·80		0·62
**CHD death or MI admission**	N = 1260	N = 1260		N = 1081	N = 1110	N = 1260	N = 1260
Events–no (%)	90 (7·1%)	61 (4·8%)		146 (13·5%)	138 (12·4%)	236 (18·7%)	199 (15·8%)
Hazard ratio (95% CI)	Referent	0·64 (0·46–0·88)		Referent	0·89 (0·71–1·13)	Referent	0·79 (0·66–0·96)
p-value		0·007			0·34		0·016
**CHD death or CHD admission**	N = 1260	N = 1260		N = 1031	N = 1061	N = 1260	N = 1260
Events–no (%)	143 (11·3%)	111 (8·8%)		197 (19·1%)	186 (17·5%)	340 (27·0%)	297 (23·6%)
Hazard ratio (95% CI)	Referent	0·74 (0·58–0·95)		Referent	0·86 (0·70–1·05)	Referent	0·81 (0·69–0·95)
p-value		0·019			0·14		0·0081

### Sub-group analyses (Scottish cohort only)

**Figure 3:**
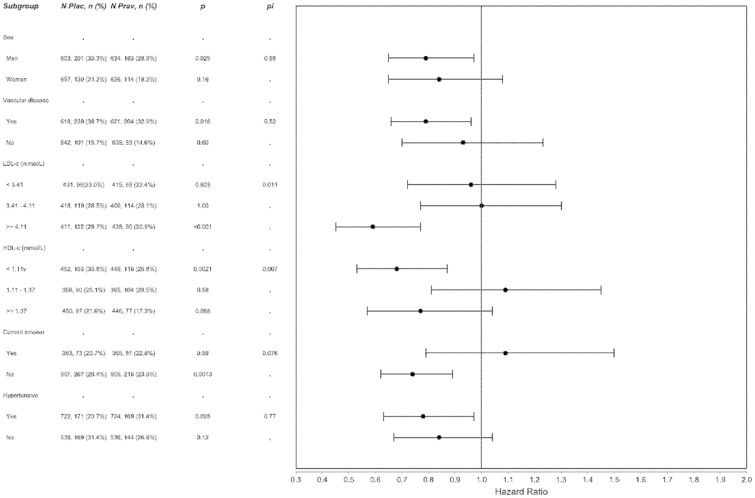
Hazard ratios and 95% CIs for the treatment effect of pravastatin relative to placebo by subgroups. Table provides number of subjects, number (percentage) of events, p-value within each subgroup and p for interaction (PI) across subgroups. Models adjusted for age, sex, current smoker, histories of diabetes, hypertension, coronary disease, cerebrovascular disease and peripheral vascular disease, BMI, SBP, DBP, HDL& LDL as appropriate.

Estimated treatment effects are illustrated in Figure 3 in the pre-defined subgroups for the outcome of coronary heart disease death or coronary hospitalization. There was evidence of heterogeneity of treatment effect only in the sub-groups based on thirds of the distribution of LDL-cholesterol. There was strongest evidence of a treatment effect in the highest third of the distribution and an associated statistically significant interaction (p = 0.011).

## Discussion

Mortality and cancer outcomes were evaluated in the full cohort of participants.

### Mortality

Despite evidence of a reduction in CHD deaths, we found no evidence that treatment of older high-risk subjects with pravastatin for several years prolonged life expectancy. PROSPER did not demonstrate a reduction in mortality during the initial period of 3.2 years [3]. With extension of follow-up to an average of 8.6 years, during which a total of 2463 (42%) subjects died, there was no evidence of a reduction or an increase in mortality associated with statin treatment. Although there was a 20% reduction in coronary mortality (p = 0.0091), there was no reduction overall in cardiovascular deaths with the reduction in coronary mortality being compensated for by a trend to an increase in stroke deaths and non-stroke non-coronary cardiovascular deaths.

### Cancer

In the post-trial follow-up of PROSPER there was no excess of cancers associated with several years of prior in-trial pravastatin treatment, resulting in no excess over the full period of follow-up (p = 0.22). This is in keeping with the recent meta-analysis that showed no evidence of cancer excess in statin-treated participants within trials in the elderly [5]. Our results were consistent when minor skin cancers and neoplasms of uncertain or unknown behaviour were added to the dataset. In the original within-trial results, there was a statistically significant 25% increase in incident cancers, p = 0.020 [3]. The results achieved using record linkage are qualitatively similar (23% increase in hazard, p = 0.038). During the within-trial period the imbalance in incident cancers appeared to evolve early. For a cancer promoting treatment it would be expected that more prolonged follow-up would give rise to an increasing imbalance over time. Therefore, the extended follow-up results support the explanation that the in-trial association of pravastatin with increased cancer risk was a chance finding.

### Composite fatal or non-fatal cardiovascular events

Composite cardiovascular fatal or non-fatal events were available only in the Scottish cohort. For the outcome of coronary or stroke death, MI or stroke admission, although there was a trend to a reduction in the number of events in long-term follow-up, this did not reach statistical significance in this period or overall.

In contrast, there was a 21% reduction in the risk of coronary death or non-fatal MI (p = 0.016) over the full follow-up period. This comprised a 36% reduction in risk (p = 0.007) within-trial, and a non-significant trend (HR 0.89, 95% CI 0.71–1.13) to a reduction post trial. The number of events due to MI detected by record linkage within-trial was smaller than reported previously, as the original results included silent and unrecognised episodes of MI [3]. For CHD death or hospitalisation there was, over the full follow-up period, a 19% relative reduction in risk, 3.4% absolute reduction (p = 0.0081); this comprised a 26% reduction in risk during the trial (p = 0.019), and a non-significant trend to a reduction in follow-up. Approximately 29 participants had to be randomised to statin treatment for an average of 3.2 years to prevent one coronary death or hospitalisation over a total of 8.6 years of follow-up.

The significant interaction between baseline LDL-cholesterol levels and long-term reduction in coronary events is interesting. However, this could be a chance finding. Our results are limited to data from the Scottish cohort in contrast to the within-trial analysis in the report of the original trial involving the full cohort with coronary and stroke deaths and stroke and MI hospitalisations as the endpoint, where a significant interaction of treatment was found with baseline HDL-cholesterol level and not LDL-cholesterol.

Consistent with the within-trial results, extended follow-up of the Scottish participants found no evidence of long-term benefit for the outcome of fatal or non-fatal stroke. However, the finding is contrary to the results from other statin trials; a meta-analysis of statin trials found a reduction of 15% in stroke events for subjects treated with a statin compared to placebo [11].

### Other statin trials in the elderly

Previous statin trials including significant numbers of elderly patients include the Study Assessing Goals in the Elderly (SAGE) trial of 893 ambulatory coronary heart disease patients demonstrating significant ischaemia on ambulatory monitoring [12]; the Heart Protection Study (HPS) [13] and the Justification for the Use of statins in Prevention: an Intervention Trial Evaluating Rosuvastatin (JUPITER) [14] (5806 in HPS and 5695 in JUPITER). SAGE included only patients with coronary disease, whereas two-thirds in HPS and none in JUPITER had known coronary disease. Approximately 90% of PROSPER participants would have been excluded from JUPITER.

The Cholesterol Treatment Trialists (CTT) Collaboration reported significant risk reductions in coronary and vascular events for statin treatment versus placebo in participants below and above the age of 65 years, although with a nominally significantly lower risk reduction for coronary events in the over 65 years of age group (p = 0.01 for interaction) [4]. Similar benefits were demonstrated for a reduction in vascular events in a subsequent CTT meta-analysis which included statin vs. control and high-dose versus low-dose statin trials for the age groups <65, ≥65 to <75 and ≥75 years [11].

### Other statin trials with long-term follow-up

In the long-term follow-up of HPS to 11 years there was no additional benefit or evidence of harm [15]. However, the absolute risk reductions for vascular events achieved during the trial appeared to be sustained. Although there was a reduction in all-cause mortality during the trial in participants aged 70 years or more, there were similar mortality rates post-trial in the two treatment groups and overall a statistically significant reduction in mortality was maintained The Anglo-Scandinavian Cardiac Outcomes Trial–Lipid lowering arm (ASCOT-LLA) investigators reported long-term follow-up of deaths to a median of 11 years in a primary prevention trial of hypertensives [16]. Although there was no evidence of a reduction in mortality within-trial, there was a nominally statistically significant reduction in deaths post-trial and overall (both p = 0.02). Surprisingly, this seemed to be associated with a reduction in non-cardiovascular deaths. The Long-term Intervention with Pravastatin in Ischaemic Heart Disease (LIPID) investigators extended follow-up to 8 years [17]. In the additional two years there was further reduction in all-cause mortality (p = 0.029) and CHD mortality (p = 0.026). In the Scandinavian Simvastatin Survival Study (4S), extended follow-up of approximately two years resulted in more deaths in the extension in the group originally assigned to simvastatin compared to the placebo group [18]. Nevertheless, over the entire period of follow-up, there remained a 30% reduction in all-cause mortality. The West of Scotland Coronary Prevention Study (WOSCOPS) reported results on ten years extended follow-up in a primary prevention trial of pravastatin versus placebo [6]. There was evidence of sustained and ongoing reduction in coronary events and at fifteen years post-randomisation there was an overall reduction in all-cause mortality and stroke outcomes. The results of these extended follow-up studies are consistent in that there was no evidence of evolving harm and the benefits accrued during the trials were, at least, sustained long-term. The results for PROSPER fit with this general pattern. In WOSCOPS where follow-up was much longer and many participants were untreated with a statin post-trial in contrast to the secondary prevention trials, there was clear evidence of ongoing benefit beyond the initial trial period providing the ability to demonstrate that relatively short term treatment (five years) can provide very long term benefit.

### Limitations of the current study

We do not know the treatment status of the participants post-trial. All participants had the opportunity to be unblinded at the end of the trial to their randomised treatment. This could have affected subsequent treatment. However, in WOSCOPS, we have previously shown that future treatment with statins was not strongly influenced by original study randomised treatment status [6]. In PROSPER, the within-trial treatment period was only 3.2 years on average and treatment was with a drug (pravastatin) that is less effective in reducing LDL-cholesterol compared to other commonly used statins. Short term and longer term treatment benefits might have been greater with more prolonged statin administration and/or treatment with a statin that would result in greater reductions in LDL-cholesterol.

### Concluding remarks

Treatment of a mixed group of elderly subjects with or at risk of vascular disease for 3.2 years with pravastatin gives long-term protection against coronary heart disease but does not prolong life. Reassuringly, this drug treatment is not associated with any long-term increased risk of cancer. It appears that the long-term CHD mortality benefits from pravastatin in older people are modest, and that the effects of competing causes of mortality result in no long-term survival gains. In comparison to the significant and apparently long term ongoing benefits of statin treatment observed in the WOSCOPS trial of 45–65 year old participants, these results underline the importance of intervening earlier in life prior to symptomatic disease onset to provide the greatest individual and public health benefits.

## Supporting Information

Table S1 **Baseline characteristics of PROSPER cohorts by treatment allocation–full cohort & Scottish cohort.**
(DOCX)
