# Supplementary material for: Long-Term Effects of Statin Treatment in Elderly People: Extended Follow-Up of the PROspective Study of Pravastatin in the Elderly at Risk (PROSPER)
Source: PLoS One. 2013 Sep 2;8(9):e72642. doi: 10.1371/journal.pone.0072642 (PMC3759378; doi:10.1371/journal.pone.0072642)
Supplement: Table S1 — Baseline characteristics of PROSPER cohorts by treatment allocation–full cohort & Scottish cohort. (DOCX) [file pone.0072642.s001.docx]

| **Table S1: Baseline characteristics of PROSPER cohorts by treatment allocation - full cohort & Scottish cohort** |
| --- |

|  | | | ***Full Cohort*** | | | ***Scottish Cohort*** | |
| --- | --- | --- | --- | --- | --- | --- | --- |
|  | | | ***Placebo*** | ***Pravastatin*** | ***Placebo*** | | ***Pravastatin*** |
|  | | | ***(n = 2913)*** | ***(n = 2891)*** | ***(n = 1260)*** | | ***(n = 1260)*** |
| **Continuous variates [mean (SD)]** | | |  |  |  | |  |
| Age (years) | | | 75·3 (3·4) | 75·4 (3·3) | 75·3 (3·4) | | 75·4 (3·4) |
| Systolic Blood Pressure (mmHg) | | | 155 (22) | 155 (22) | 154 (22) | | 153 (21) |
| Diastolic Blood Pressure (mmHg) | | | 84 (12) | 84 (11) | 83 (11) | | 83 (11) |
| Height (m) | | | 1·7 (0·1) | 1·7 (0·1) | 1·6 (0·1) | | 1·6 (0·1) |
| Weight (kg) | | | 73·4 (13·5) | 73·4 (13·3) | 71·4 (12·9) | | 72·3 (13·3) |
| Body Mass Index (kg/m) | | | 26·8 (4·3) | 26·8 (4·1) | 26·7 (4·2) | | 26·8 (4·2) |
| Total cholesterol (mmol/L) | | | 5·7 (0·9) | 5·7 (0·9) | 5·7 (0·9) | | 5·7 (1·0) |
| LDL cholesterol (mmol/L) | | | 3·8 (0·8) | 3·8 (0·8) | 3·8 (0·8) | | 3·8 (0·8) |
| HDL cholesterol (mmol/L) | | | 1·3 (0·3) | 1·3 (0·4) | 1·3 (0·4) | | 1·3 (0·4) |
| Triglycerides (mmol/L) | | | 1·5 (0·7) | 1·5 (0·7) | 1·6 (0·7) | | 1·6 (0·7) |
| **Categorical variates [n ( %)]** | | |  |  |  | |  |
| Men | | | 1408 (48%) | 1396 (48%) | 603 (48%) | | 634 (50%) |
| Current smoker | | | 805 (28%) | 753 (26%) | 353 (28%) | | 355 (28%) |
| Medical History | | |  |  |  | |  |
|  | -Diabetes | | 320 (11%) | 303 (10%) | 112 (9%) | | 101 (8%) |
|  | -Hypertension | | 1793 (62%) | 1799 (62%) | 722 (57%) | | 724 (57%) |
|  | -Vascular disease | | 1259 (43%) | 1306 (45%) | 618 (49%) | | 621 (49%) |
|  |  | -Peripheral vascular disease | 311 (11%) | 338 (12%) | 155 (12%) | | 168 (13%) |
|  |  | -Coronary heart disease | 916 (31%) | 965 (33%) | 473 (38%) | | 477 (38%) |
|  |  | -Cerebrovascular disease | 324 (11%) | 331 (11%) | 135 (11%) | | 131 (10%) |
